# “Desired behaviors”: alignment and the emergence of a machine learning ethics

**DOI:** 10.1007/s00146-025-02272-3

**Published:** 2025-03-21

**Authors:** Katia Schwerzmann, Alexander Campolo

**Affiliations:** 1 Project Interact! New Forms of Social Interaction with Intelligent Systems, Ruhr-Universität Bochum, Universitätsstr. 150, Bochum, 44801 Germany; 2 Department of Geography, Durham University, Durham, Lower Mountjoy, South Road, DH1 3LE UK

**Keywords:** Alignment, Norms, Values, Machine learning, Artificial intelligence, Behaviors

## Abstract

The concept of alignment has undergone a remarkable rise in recent years to take center stage in the ethics of artificial intelligence. There are now numerous philosophical studies of the values that should be used in this ethical framework as well as a technical literature operationalizing these values in machine learning models. This article takes a step back to address a more basic set of critical questions: Where has the ethical imperative of alignment come from? What is the ethical logic of alignment—how, exactly, does it propose to regulate machines’ and peoples’ conduct? And what are the social and political implications of this ethics? After discussing the logical and normative implications of the term itself—in what sense alignment can have an ethical meaning—we undertake a four-part “anatomy” of alignment in contemporary large language models (LLMs): first, a relatively technical sense in sequence modeling; second, a more normative sense relating to how outputs of pre-trained models are ethically evaluated; a third sense where external values are introduced using fine-tuning techniques to manage undesired model behaviors; and fourth sense, where alignment is given extreme ethical stakes in philosophical discussions of existential risks. We find that the ethics of alignment is fundamentally concerned with the problem of control, with unintended model behaviors that arise from divergences between training objectives and the normative expectations that govern the contexts in which they are used. Alignment serves to bridge the gap between what we call an “is” normativity, of statistical patterns identified by models and an “ought” normativity where values are technically introduced in models to steer them away from undesired behaviors. By problematizing control, the ethics of alignment weakens capacities to both make more substantive ethical judgments and also political decisions about how to live with AI.

## A new ethical principle

Over the past decade, a new concept has appeared in the ethics of artificial intelligence: alignment. One notable early instance was in the “Asilomar AI Principles,” issued in 2017 by an organization called The Future of Life Institute. Its authors affirm: “Highly autonomous AI systems should be designed so that their goals and behaviors can be assured to align with human values throughout their operation” (Future of Life Institute 2017). By 2020, this word was commonly used, for instance as the title of a book by the journalist Brian Christian: *The Alignment Problem: Machine Learning and Human Values* (2020).

More recently, the idea of alignment has become closely associated first with large language models (LLMs) and now other types of generative models, such as multimodal (Amirloo et al., 2024) and text-to-image models (Lee et al. 2023; Wu et al., 2023). For instance, in 2022, a group of researchers based at OpenAI wrote:

Large language models (LMs) can be “prompted” to perform a range of natural language processing (NLP) tasks, given some examples of the task as input. However, these models often express unintended behaviors such as making up facts, generating biased or toxic text, or simply not following user instructions. . . This is because the language modeling objective used for many recent large LMs—predicting the next token on a webpage from the internet—is different from the objective “follow the user’s instructions helpfully and safely”. . . Thus, we say that the language modeling objective is misaligned. Averting these unintended behaviors is especially important for language models that are deployed and used in hundreds of applications (Ouyang et al. 2022, pp. 1–2).

This statement about LLMs “undesirable behaviors” (Ouyang et al. 2022, p. 20) captures specific technical contexts of alignment, while also gesturing to deeper philosophical problems, like the interpretation of intentions and the complexity of clarifying what humans deem desirable when it comes to machines. These senses resonate with a statement made by Norbert Wiener in 1960, often referenced in the alignment literature: “If we use, to achieve our purposes, a mechanical agency with whose operation we cannot efficiently interfere once we have started it, because the action is so fast and irrevocable that we have not the data to intervene before the action is complete, then we had better be quite sure that the purpose put into the machine is *the purpose which we really desire and not merely a colorful imitation of it (our emphasis)*” (Wiener 1960, p. 1358). How the machine learning community conceptualizes, agrees upon, and technically operationalizes AI’s “desired behavior” (Ouyang et al. 2022, p. 4) is at the heart of the alignment problem.

In this essay, we critically study alignment as an emerging ethical framework for artificial intelligence, one that in the past decade has become something of a common sense in both technological and philosophical communities. Much of this literature can be divided into normative debates about the types of human values to which AI systems should be aligned (Floridi et al. 2018; Christian 2020; Gabriel 2020; Gabriel and Ghazavi 2021; Han et al. 2022; Kasirzadeh and Gabriel 2023; Raper 2024) or techniques for somehow encoding these values in AI systems (Cho et al. 2015; Askell et al. 2021; Ouyang et al. 2022; Bai et al. 2022a, b; Glaese et al. 2022; OpenAI 2023; Casper et al. 2023; Gulcehre et al. 2023; Ngo et al. 2023). Both strands assume an ethical need for *alignment* as more or less self-evident, a demand imposed on us by the nature of the machine learning systems.

Our article begins instead by asking where this ubiquitous ethical imperative for alignment has come from. What has made it such a self-evident, even hegemonic approach to governing contemporary AI? How, precisely, does it guide ethical conduct, the behaviors of both machine learning models and the human beings subjected to them? And beyond the sphere of ethics, what forms of politics does alignment open onto?

To answer these questions, we undertake an “anatomy” of alignment. Crawford and Joler (2018) have used this term to describe the complex interaction of geographies, data, materials, and labor that constitute AI systems. Our own reference to anatomy focuses more on the way “alignment” operates across different technical-discursive levels (although these depend on the systems that Crawford and Joler anatomize). Alignment’s recent emergence, to use Michel Foucault’s language, constitutes an “event,” in which the uses of this term come together into a distinctive conceptual relationship, albeit without referring to any pre-given object or “whole” (Foucault 1972, p. 56). These contexts range from abstract normative philosophy to the concrete, technical ways that alignment is practiced by different machine learning communities.

We take alignment as *neither* a technologically determined necessity (the view of some proponents) *nor* some pure ideology imposed from the outside to obfuscate the real effects of machine learning systems (the view of some critics). Rather, our anatomy of alignment foregrounds its movement across these very different registers, paying special attention to the technical aspects of machine learning models that lead to the sorts of unintended or undesirable behaviors to which alignment can be offered as an ethical corrective. While we sometimes agree with critics who dismiss these ethical frameworks as distractions from more pressing issues of justice (Greene et al. 2019), we also believe it is worth studying the emergence of alignment seriously for what it can tell us about the way the machine learning community frames in ethical terms political issues of power and resource asymmetries, and of “interpretative sovereignty” (*Deutungshoheit*)^1^ by determining what relationship to AI is “desirable” for society. Throughout this article, we call into question a received division of labor between technical and ethical approaches, where the choice of values can be left to different groups or individuals and simply instantiated by engineers through alignment techniques. Instead, we want to show how different forms of normativity permeate these levels, interacting to produce a distinctive way of regulating conduct.

We begin with a brief discussion of the concept “alignment” in a more general sense, before describing four interconnected anatomical levels, related to LLMs. On a first level corresponding to an early technical usage, alignment refers to the relation between the structure of inputs and outputs in sequence modeling. A second level refers to a form of normativity produced by pre-training objectives, which identify patterns expressing “the natural structure of [the data]” (Chollet 2021, 141), such as the proximity, in the input space, between specific tokens (e.g. the fact that “she”, “he”, “it” is followed by “is” rather than “are”). We refer to this form of quantitative, statistical normativity as an “is” normativity.

The third level refers to fine-tuning as a set of technical methods that operationalize ethical values to mitigate these undesirable and unexpected model behaviors, sometimes in collaboration with academic philosophers, a form of normativity that we call an “ought” normativity. These qualitative values are subject to quantitative forms of maximization through reinforcement learning algorithms like proximal policy optimization (PPO) (Schulman et al. 2017) or direct preference optimization (DPO) (Rafailov et al. 2024).

The fourth level is situated outside of these programming practices; here alignment becomes a speculative philosophical framework that moves into the extreme normative territory of existential risks, or the potential end of humankind. At this final level, more concrete interactions grounded in normative and technical practices recede in favor of a much more speculative ethical vision.

These layers form a kind of ethics in which the control of (model) behaviors is fundamentally problematic. Alignment conjures a world of unintended consequences, unintelligible, high-dimensional spaces of prediction and action. “Behaviors”—of models and humans—cannot be prescribed by universal rules of ethical conduct, nor can effects be easily calculated in advance according to consequentialist principles, although the latter certainly informs some of the extreme existential ideas concerning alignment. It is an ethics that speaks to an unpredictable world that cannot be effectively regulated by familiar deontological and consequentialist ethics. Rather, values or preferences are encoded during a fine-tuning phase in order to elicit (rather than directly prescribe) desirable behaviors from both humans and models, while steering away from unanticipated and undesired ones. These layered discourses lead to tensions in the ethics of alignment.

On the one hand, alignment ethics presents itself as humble, promulgating minimal values (such as truthfulness, harmlessness, and helpfulness) that command broad assent in liberal societies, in order to mitigate machines’ undesirable behaviors. On the other hand, alignment is oriented toward horizons of the so-called “existential risks” of species extinction that would be caused by machines getting out of control.^2^ Alignment also represents de facto a massive project of normativization that regulates not only machines’ behaviors but also society’s relationship with AI, naturalizing a set of contingent, partial conventions, such as a dialog with a chatbot, where knowledge is presented in an easily digestible, “neutral” bullet point format, or generated images centered around whiteness, photographic realism, the heteronormative family, etc.

These tensions have the paradoxical effect of associating the most advanced technologies with a minimalist, disempowering form of ethics, in which the political ability to shape a common world recedes under the shadow of potentially ungovernable machine learning models. It is not a coincidence that alignment ethics is promoted most enthusiastically by precisely the technology companies who stand to profit from a globalized standardization of norms and values around AI. Alignment has become a geopolitical as well as an ethical and engineering watchword (European Union 2023, p. 11). What our analysis makes clear is that even a minimal ethics doesn’t escape issues of power. Instead, we argue that the framework of alignment technologically pacifies conflicts over values, hides the interpretative sovereignty at play in deciding what behaviors are desirable and how society may want to shape its relationship to AI. The practice of alignment excludes these issues from the sphere of democratic debate and decision-making.

## Alignment as concept

### Technology and normativity

Before analyzing alignment more directly, it is necessary to introduce our diagnostic approach to ethics, norms, and machine learning technologies. When we speak about norms, we understand them as both means of guiding actions and representations of how things could or should be (Foucault 2018, p. 214). Norms embody and operationalize values, which are themselves qualitative interpretations of the world as something that concerns humans (Nietzsche 1974, pp. 241–242). Following the philosopher Georges Canguilhem, norms gain their meaning and guiding force precisely when expectations aren’t met, when a “disappointing” state of affairs must be replaced by a “satisfying” one (1991, p. 240).

Much of the existing critical literature on bias and fairness in machine learning concerns what we call an “is” normativity—the way that models identify certain structures in their training data.^3^ There is a long philosophical tradition, often dated to David Hume, that makes strong distinctions between the factual “is” and the normative “ought” (2007, 302). Our use of the term “is” works against this grain. An “is” normativity should not be taken as an endorsement of the naive idea that training data is some naturalistic or neutral reflection of the phenomena that it represents. As many critical scholars have shown, this data, selected and aggregated through labor-intensive processes, is the product of relations of power—of racism, sexism, or other forms of domination in the societies that produced it. Machine learning further intensifies this form of normativity when large datasets undergo both *curation* and *feature engineering* to optimize models’ ability to learn from them—a process we previously called “exemplification” (Campolo and Schwerzmann 2023). Our association of “is” and “normativity,” of fact and value, description and prescription, is precisely meant to draw attention the *tension* between the models’ ability to extract statistical patterns that reflect “the natural structure of [the] data” (Chollet 2021, p. 141) on which they were trained and the fact that the data itself is the product of cultural norms and valuations and that the patterns must be made discoverable through a complex process of exemplification.^4^ Furthermore, it serves to analytically differentiate this form of normativity from another, explicit and openly-assumed form of normativity that is characteristic of alignment ethics.

What we term an “ought” normativity in alignment represents a response to the ethical problem of the “is” normativity. It recognizes that statistical “is” norms learned during pre-training can often “disappoint” users or violate their implicit normative expectations. This “ought” normativity—introduced during the supervised phase of fine-tuning—works to mitigate such undesirable model (and user) behaviors by specifying values that guide a subsequent reinforcement learning phase. These more recognizably ethical “oughts”—for instance, a model ought to provide truthful responses—cannot, however, be specified directly because of machine learning’s exemplary programming paradigm (Campolo and Schwerzmann 2023). Instead, values such as “harmlessness” are used to guide the production of human-curated examples that are used to produce a new, fine-tuned model. In sum, this “ought” normativity seeks to mitigate divergences between model behaviors and normative expectations by naming “values” that counteract patterns of undesired behaviors (such as untruthful outputs), and are used to produce examples that guide the model toward desired behaviors.

Our fundamental hypothesis is that alignment ethics seeks to domesticate the tension between the “is” and the “ought” normativity, to resolve the divergences between statistical patterns learned from the training data and normative expectations for how models should behave—in a world where direct control over their behaviors is problematic.

### Logics of alignment

Still, the question remains, *how* does alignment work to calibrate statistical norms and desired behaviors? The term draws from sedimented meanings and connotations that precede its use in the context of machine learning. The *Oxford English Dictionary* refers to both the action and result of “arranging in or along a line, or into appropriate relative positions”; the action and result “of bringing into line; an instance of this” (*OED* 2024). The implicit normative dimension resides in the criteria by which relative positions can be established as “appropriate.” We note that there are two possible actualizations of alignment understood *as a mode of relation that put relata in their appropriate position*: a relative and an absolute one, which has a bearing on technical implementations of values.

Logically, there is a first symmetrical, “relative” sense of alignment: when elements take their “appropriate” position relative to each other. This logic is captured by an old militaristic sense of alignment as falling into line or formation, whereby soldiers arrange themselves in relation to both some abstract representation of an order and the others around them. We argue below that this sense of alignment corresponds to the engineering practice of aligning a model through fine-tuning. In this process of mutual rapprochement, humans align with machines in the sense that they adopt the ethics and formulate the values that are encodable in machines; and machines align with humans by “learning” these values.

This logic contrasts with an asymmetric, “absolute” sense of alignment, where the individual elements are subordinated to a fixed, determinate, hegemonic position to which they align—rather than to each other. We might think here of the geopolitical category of aligned vs. non-aligned countries that emerged during the Cold War, where postcolonial countries refused to submit to the polarized binary between eastern and western, communist and capitalist blocs. We sense that this second, asymmetric logic of alignment permeates existential risk discourses, developed among others by the philosopher Nick Bostrom (Bostrom 2013, 2014; Dung 2023). In these cases, alignment refers to the asymmetry between a human will and intellect framed as finite and the speculatively posited “superintelligence” of AI. Either machines are made to submit to human values and expectations, or, if machines were left to their own devices, humanity could face catastrophic extinction.

While in practice, these two logics of alignment cannot easily be kept separate, the purpose of the distinction is to provide a framework to understand different types of discourse on alignment. In both logics, we notice that alignment is never only about values and always also about power, more specifically the power to bring different entities into an ethical relation that can be symmetric or asymmetric. Thus, alignment must be considered not only as an ethical but also as a political question. We hypothesize that the framework of alignment has so far led to the formulation of the political issue of power over models through an exclusively ethical vocabulary that seeks to technically solve a problem that is always more than ethico-technical, one that necessarily exceeds any particular alignment technique: the problem of who may decide on the fundamental positions to which values and behaviors must be oriented.

## An anatomy of alignment

### Sequence modeling in natural language processing

One reason that the term “alignment” has resonated strongly within the machine learning community, is that it has been used to conceptualize a class of technical problems in natural language processing (NLP): structured output problems. For instance, in Cho et al.’s 2015 paper, “Describing Multimedia Content Using Attention-Based Encoder-Decoder Networks,” the use of the word “alignment” resonates with the sense of “bringing into appropriate relative positions.” In structured output problems like machine translation, the structure of the output is “somehow related” to “the structure of the input” (2015, p. 1875). Here, the problem of alignment refers to how subcomponents of the input should be related to subcomponents of the output; in other words, how words and grammatical relationships in the source sentence align with those in the target sentence. The question is how to model this relation.

What is at stake in the question of alignment in sequence modeling exceeds simple classification problems. Indeed, sequence modeling problems go beyond learning a direct mapping of input to output such as between an image and a label. In translation problems both the input and the output are syntactically complex, with each element contextually dependent on the others; so the question of alignment concerns the optimal mapping of a structure onto another—a mapping of the probability distribution of the subcomponents of the output to the probability distribution of the subcomponents of the input (Cho et al. 2015, p. 1875). So-called attention mechanisms (Bahdanau et al. 2016) play an essential role in arranging elements in the desired relative position by allowing the model to attend to each element of the input while weighing their importance relative to all the other elements. This “attention score” allows for a more accurate prediction of the relevance and order of the components of the output. In image caption generation, to take another example, sequence modeling means aligning the spatiality of the image or the spatio-temporality of the video with the linearity of the caption in order “to accurately describe the spatial relationships between elements of the scene represented in the image” (Cho et al. 2015, p. 1875).

Thus far, the language of alignment in sequence modeling problems is principally technical, even formal. It draws on the existing “relative” sense of alignment in that it concerns correspondence or parallelism between structured input and output. The alignment problem here concerns the “is” normativity of the training data, reflected in the statistical patterns learned by the model, its “representations.”

### Model pre-training: the case of hallucination

How, then, do explicitly named “human values” enter this picture? At a very high level, this is due to the simple and inescapable fact that machine learning models are used in social contexts shaped by diverse normative expectations, where statistically-derived outputs are interpreted and evaluated ethically, as seen in well-founded concerns about biased outputs (Blodgett et al. 2020).^5^ However, the case of large language models shows that there are more specific, technical pathways leading to the sorts of unintended consequences or undesirable behaviors to which alignment is proposed as an ethical corrective. Most broadly, what interests us is how the *divergences* between training objectives and downstream model behaviors, or, more precisely, human evaluations of these divergences have been fashioned into an ethico-technical problem, that of alignment. Ouyang et al.’s statement quoted at the outset of this article encapsulates this more specific technical pathway. Alignment problems occur “because the language modeling objective used for LLMs—predicting the next token on a webpage from the internet—is different from the objective ‘follow the user’s instructions helpfully and safely’” (Ouyang et al. 2022, p. 2). While many critics have focused on limitations of the statistical nature of the language modeling objective (Bender et al. 2021), what Ouyang et al.’s statement shows is that it is not so much the statistical nature of the objective itself that produces alignment problems (although this may of course be problematic in its own right) but rather gaps or divergences between this training objective and ethical expectations about how models should behave.

This language modeling objective is more precisely a pre-training objective, and it is this paradigm, in which a single training objective is used for a wide range of tasks in downstream social contexts that best illustrates how alignment problems arise in generative AI models. The idea of pre-training is not especially new. It constitutes one of the conceptual innovations at the heart of the deep learning renaissance. The technique emerged in response to one of the optimization problems raised by deep neural networks: “initialization”—how should one set the initial values of a very large number of parameters in a network to achieve some acceptable level of performance? One ingenious strategy is to begin with a generative, unsupervised training algorithm that learns a rough shape of the input distribution, which can be used as an initial set of weights for supervised learning on more specific tasks (Hinton et al. 2006; Bengio et al. 2007). This strategy was especially effective in domains like sequence modeling, which faced the problem of huge, unlabeled corpora of textual input data from the internet (Dai and Le 2015).

Subsequent architectural innovations, notably the transformer (Vaswani 2017 et al.), in concert with much larger training datasets and model sizes have expanded ideas about pre-training from an initialization technique into a full-blown training paradigm for LLMs, with the goal of learning “a universal representation that transfers with little adaptation to a wide range of tasks” (Radford et al. 2018, p. 2). However, researchers soon noted that unexpected, undesirable behaviors may arise during this process of transfer. Take the well-known issue of “hallucination” in generative models. The metaphorical use of a charged psychological term—referring to a vivid or realistic perception in the absence of appropriate external stimulus—indicates the difficulty of defining such problems in a value-neutral way (Förster and Skop *forthcoming*).^6^ In the context of LLMs, “hallucination” refers to the production of text that “gives the impression of being fluent and natural despite being unfaithful [with respect to inputs] and nonsensical. It appears to be grounded in the real context provided, although it is actually hard to specify the existence of such contexts”—or more colloquially the tendency of language models to make things up (Ji et al. 2023, p. 2). Needless to say, such formulations beg further questions about the nature of linguistic reference and even truth.

We must bracket these issues and focus on how hallucination became intelligible first as a *specific type of divergence between model output and desired behavior* and then a negative evaluation of this divergence, expressed in pathologizing language. Although the problem of hallucination may now seem obvious and even intractable, its formulation illuminates the necessarily retrospective character of alignment’s ethical judgments, where courses of action cannot be taken in advance according to any deontological principle or calculation of utility but rather *emerge* through evaluations of unintended behaviors, which become intelligible in light of gaps between broad training objectives and narrower tasks. The specification of values or preferences responds to these undesired behaviors and attempts to mitigate them, transforming them into (retrospectively) desired ones.

In fact, these types of divergences between a probabilistic language modeling objective and model outputs were recognized earlier, as in the case of “degeneration” of text produced by language models (Welleck et al. 2019), where “the use of likelihood as training objective leads to…output text that is bland, incoherent, or gets stuck in repetitive loops” (Holtzman et al. 2020, p. 1). In another task, that of document summarization, this problem was reformulated as text that is “unfaithful” to a source document, leading to the use of the term hallucination (Maynez et al. 2020). Ironically, recognition of this problem is only possible due to the remarkable ability of language models, trained on huge amounts of text from the internet, to store or compress information in their parameters, leading people to develop expectations that they draw on a corpus of presumably factual “world knowledge” from which probabilistically generated outputs can diverge. These researchers also perceptively noted that for more open-ended tasks like conversations, models are *required* to hallucinate in some sense when they produce textual outputs not directly implied by or inferrable from the input (Maynez et al. 2020, pp. 1907–1909).

The case of hallucination illustrates how the “is” form of normativity that emerges out of the specific pre-training paradigm of large language models—predict the next most likely token given some previous sequence of tokens—interacts with the “ought” normativity of researchers’ and users’ expectations about how linguistic interactions should take place. These gaps have proven difficult to predict in advance, a fact that speaks to the more general sense that alignment is an ethics for a world of undesired behaviors, where apparently benign but necessarily underspecified training objectives can yield unpredictable results when models are released into the world. The case of hallucination shows that certain types of likelihood-based model behaviors are retrospectively *evaluated* in light of the normative expectations of these contexts—in this case something like the imperative that models should respond truthfully to prompts. At this point, the question arises, how is it possible to act ethically, to govern both models and ourselves when they elude our intentions and attempts at control? How might external norms, “oughts,” step in to smooth or domesticate these divergences?

### Fine-tuning

Misalignments like hallucination are not, then, the result of model errors in a strict sense. If they are described in pathological terms, it is instead because they “disappoint” users’ expectations, to use Canguilhem’s vocabulary. What alignment techniques in generative models work to bridge is precisely the gap between pre-training objectives, the source of “is” normativity, and normative expectations (the “oughts”).

Alignment as an explicitly ethical problem is addressed at the stage of fine-tuning, or when pre-trained models are adapted to more specific tasks like answering prompts or dialoguing with the user. One prominent set of techniques used during fine-tuning is called “reinforcement learning from human feedback” or RHLF. It involves—in the case of InstructGPT laid out in Ouyang et al. (2022)—first, the selection of human labelers to both produce and ethically evaluate examples, second, the technological encoding of values in examples used to train a “policy” with the help of a “reward model,” and third, the choice and operationalization of these values.

Before the fine-tuning process starts, InstructGPT labelers were selected through a screening process for their “high propensity to detect and respond to sensitive content” (Ouyang et al. 2022, p. 36). This expression points toward a first, implicit form of alignment: labelers must be able not only to fulfill the task of writing prompts but also to *understand* and *agree* with the researchers’ judgments and the company’s values in reference to which examples must be produced and evaluated (Ouyang et al. 2022, p. 36). This assignment is more complex than other, more factual data annotation tasks, like labeling images—although we stress that even the most apparently objective labeling tasks tend to involve selection and forms of evaluation. Here the labelers have to evaluate outputs following complex instructions but also rely on their intuition and sensibility (Bai et al. 2022a, p. 4).^7^ While some tasks are solved through simple fact-checking, most of them appeal to complex levels of contextualized knowledge and situational understanding. For instance, what counts as a moral judgment (Ouyang et al. 2022, p. 39) or when an output is an opinion depends crucially on the ability of the labeler to evaluate not so much the explicit content of the output (*what*) but the way it is formulated (*how*). For the model InstructGPT, OpenAI hired 40 contractors to do this labeling work (Ouyang et al. 2022, p. 2). This number seems incredibly low given the impact that this new set of aligned examples have on the fine-tuning of the entire model. 

The core technical problem, then, is how to incorporate these human evaluations into a semi-automated training process that allows the model to evaluate a much wider range of future outputs. The goal of RLHF is to train a reward model that favors the outputs that *would be preferred* by humans. This frequent slippage between preferences or desires and ethics is characteristic of the alignment framework. The process relies on several feedback loops. Following Ouyang et al., human labelers write a “desirable” output for some human written prompt as a first step. Here, we note the constitutive and yet never clearly defined normativity of what makes a desirable output—labelers presumably know them when they see them. The pre-trained model is then re-trained on the pairs of human-written prompts and answers, resulting in a new model called the “supervised learning baseline,” which produces its own set of answers to new questions. Once again, humans are employed, this time to rank the answers on some numerical scale, here 1–7, for their overall quality. In addition, the labelers have to answer “yes” or “no” to the questions: “Output A: *Fails* to follow the correct instruction/task? Inappropriate for customer assistant? Contains sexual content; Contains violent content; Encourages or fails to discourage violence/abuse/terrorism/self-harm; Denigrates a protected class; Gives harmful advice? Expresses moral judgment” (Ouyang et al. 2022, p. 39).

These ranked responses are then used to train a reward model whose function is to predict the output that would be preferred by humans *in the absence of human judgment*. Once the reward model is trained, it assigns a numerical score to the desirability of the output and is used to optimize a policy (PPO). A policy is a reinforcement learning model that contains the “strategy” based on which the model produces output that yields maximum reward. This strategy is recursively optimized in accordance with the reward model’s feedback. Finally, the optimized policy is used to update the supervised learning baseline, leading to the fine-tuned model.

While the literature on reinforcement learning is replete with the vocabulary of “goal” and “objective,” these are never final, in the sense of something toward which the model and the society in which it is embedded should strive (like values, such as the common good, justice, freedom). Instead, goals and objectives are always recursively instrumentalized as proxies, that is, *as means* to quantify the potential for future optimization: the goal of fine-tuning is in the abstract “alignment,” but alignment, concretely, is a means to score best “for now” on benchmarks compared to other models. In machine learning, training goals always recursively turn into instrumental means. In that sense, alignment is a minimalist kind of ethics—a minimalism that makes it possible to adapt to many different tasks or uses.

What kind of guidance is given to human labelers to produce and correctly evaluate examples? How is this ethical “feedback” created and used to produce examples capable of eliciting the desired machine behavior? In our review of the computer science literature, this is where we see professionally-trained philosophers employed by big tech companies—such as Amanda Askell (Anthropic) and Iason Gabriel (DeepMind)—enter the alignment conversation. There appears to be a consensus in the machine learning community, at least provisionally or pragmatically, about a specific set of ethical criteria or values that annotators use to evaluate examples: harmlessness, helpfulness, and honesty. In a 2021 paper, Askell et al. justify these values pragmatically: “We chose ‘helpful, honest, and harmless’ [HHH] as criteria because they are simple and memorable, and seem to capture the majority of what we want from an aligned AI” (Askell et al. 2021, p. 4). We note the circularity of this justification. Anthropic’s pragmatic choice of values has since been adopted by others in the machine learning community with some minor modifications: OpenAI has substituted honesty for truthfulness (Ouyang et al. 2022, p. 10) and DeepMind honesty for correctness (Glaese et al. 2022, p. 2). A value like harmlessness recalls the principles or norms of “non-maleficence” central to biomedical ethics (Beauchamp and Childress 2013, p. 17; Floridi et al. 2018, p. 697).^8^ Helpfulness, on the other hand, seems specific to our expectations toward what AI tools and technologies should be.

The authors of these papers recognize that there may be deep and intractable conflicts among these values or that other values outside of the HHH framework may be desirable. Additionally, these three values are highly dependent on one another such that there are often tradeoffs between them: an output can be true and helpful and yet also harmful. Alignment ethics is therefore not one of absolute principles with a claim to general validity, but one based on the necessity to compute tradeoffs between values because of their potential “conflicts” depending on whose agent the model has to be aligned with (Askell et al. 2021, p. 45). Alignment ethics thus consists in optimizing the relation between risk and reward.

To measure a model’s level of alignment, the HHH values must be turned into proxies. This enables programmers to say that, for instance, one model performs better than another on a harmfulness benchmark such as the RealToxicityPrompts dataset (Ouyang et al. 2022, p. 45). These special datasets allow the researchers to quantify qualitative values, thus turning values into norms for the regulation of model and user behaviors.

In the practice of fine-tuning, alignment follows the *relative logic* we described above*,* where humans and machines position themselves relatively to each other: humans formulate values that are encodable in machines; and machines align with humans by “learning” these values from examples formulated by humans. Conversely, humans are trained to act in a way that complies with alignment ethics. For instance, when a human asks a question deemed problematic, the model “should politely refuse” to answer (Askell et al. 2021, p. 5). One can thus describe this relative sense of alignment as a mutual rapprochement, and even domestication between humans and machines.

Our analysis of alignment as an ethico-technical solution to negotiating the gap between *is* and *ought* normativity shows the complexity of introducing norms into the model due to machine learning’s special form of normativity. In previous work (Campolo and Schwerzmann 2023), we argued that the transition from the rule-based programming paradigm of the previous expert systems to the example-based programming paradigm of machine learning brought forth a specific type of authority, that is, a specific way of “conducting the conducts” (Foucault 2008, p. 186) of both machines and humans. In contrast to Max Weber’s analysis of a rational, bureaucratic type of authority based on rules (Weber 1978), we demonstrated that machine learning enacts a specific type of authority based on examples. This type of authority regulates conducts of models and users not by the prescription of explicit programming rules but rather through norms elicited from examples, which we define as complex assemblages by which data is aggregated, formatted, and processed so that “representations” can emerge during training (Campolo and Schwerzmann 2023, p. 5). These representations express these ethico-technical norms.

Pursuing this line of inquiry, we understand alignment as a technique for managing the complexity of generative models and the undesirable character of some of their outputs by modifying their “is” normativity with the more explicit, yet still example-based “oughts,” to which model behaviors must be aligned. Given the difficulty of controlling LLMs whose abilities are ever changing and the exemplary logic of machine learning, it is not possible to codify desired behaviors as formal rules in the deterministic form of: “if this input, then that output.” Instead, norms must be learned by the model “empirically” from examples attached to norm-giving objectives. Even the recent introduction of constitutional AI (Bai et al. 2022b) or* “*rule-based reward models” [RBRM] (OpenAI 2023, p. 62) in the fine-tuning of GPT-4 does not contradict machine learning’s exemplary logic. In fact, RBRM as a reinforcement learning technique constitutes an ad hoc attempt to regulate the dynamics produced by the underlying example-based training process, increasingly through recursive feedback from the models themselves—“AI” rather than “human” feedback (Lee et al. 2024). Although the prompts formulated in natural language have a rule-like form to a human reader, in practice, they act more like labels for classifying prompts submitted to language models (Mu et al. 2024, 18).

To summarize, RLHF trains a reward model and a policy to automate desirable human judgment using values embodied in examples specifically curated for the purpose of fine-tuning. Fine-tuning can thus be described as a process of normativization of the pre-trained model. Because alignment values must cover as many potentially misaligned cases and contexts as possible, these values are minimal, even vague. They are specifiable, in retrospect, only as far as they are operationalized through example evaluations, benchmarks, and reward models.

### Existential risks

We have thus far focused on relatively technical aspects of the training paradigms of contemporary, auto-regressive LLMs. We argued that these lead to alignment problems like hallucinations due to the fact that gaps open between model behaviors generated by the general pre-training objective (“is” normativity) and normative expectations as these models are used in cultural contexts (“ought” normativity). Techniques like RLHF aim to mitigate these divergences using human evaluations of model outputs, guided by minimally specified “values,” to bridge this gap. These technical contexts are crucial for grasping complex interactions between engineering and philosophical ideas characteristic of the emerging ethics of alignment.

There is, however, another more philosophical sense of alignment that intersects with these engineering practices and ideas, relating to the possible risks posed by imagined “superintelligent” AI systems. Gaps or divergences between desired and undesired behaviors expand into chasms, even qualitative breaks between the radical finitude of human intellects when compared to the imagined capabilities of “superintelligent” AI or some other form of artificial general intelligence (AGI). We will briefly characterize the ethical claims made in this more radical, “existential” notion of alignment. Although we will focus more on the conceptual side of this ethics, we recognize that there are important institutional dimensions to this story that connect academic centers to movements, such as effective altruism and networks of wealthy donors (Goujon 2023; Burrell and Metcalf 2024).

Where did this sense of alignment come from? The phrase “alignment” or, more specifically, “value alignment” was used in a 2014 white paper written by the UC Berkeley AI scientist Stuart Russell. He argues, “for an autonomous system to be helpful to humans and to pose no unwarranted risks, it needs to align its values with those of the humans in its environment in such a way that its actions contribute to the maximization of value for the humans” (2014, p. 1). However, uses of the more specific phrase “value alignment” predate references to current artificial intelligence systems. One proximate source is a 1990s management literature associated with the systems science movement. Peter Senge, for instance, devoted a section of his influential book *The Fifth Discipline* to the idea of alignment, making it a cornerstone of his theory of learning organizations. Here, “alignment” refers to the way that individuals on a team adopt a shared sense of purpose and direction. Echoing the “relative” logic of alignment we described at the beginning of this paper, this is not the *subordination* of the individual to the team or group but rather some kind of harmonization: “individuals do not sacrifice their personal interests to the larger team vision; rather, the shared vision becomes an extension of their personal visions” (1990, p. 129). By smoothing possible divergences between individual and team interests, alignment more or less implicitly works to defuse perhaps the fundamental conflict of capitalism, that of capital (and now data) versus labor (Sadowski 2019). We can also note in passing that similar ideas about harmonizing “divergent” interests or incentives (again, implicitly defusing conflicts), for example, those of principals and agents, abound in management literatures dating back at least to the 1970s (Jensen and Meckling 1976).^9^ In a larger sense, all of these ideas speak to a desire to manage the behaviors of diverse but interdependent individuals not through some constitutive ethical decision or explicit rules but rather by shaping incentives so that their behaviors converge over time.

These conflicts or problems of divergence are radicalized by even the mere “plausibility” (Dung 2023, p. 6) of highly autonomous or “superintelligent” AI—defined non-technically as “any intellect that greatly exceeds the cognitive performance of humans in virtually all domains of interest” (Bostrom 2014, p. 22). Here divergences no longer occur between relatively similar agents but rather between totally incommensurate human and machine intelligences. According to proponents, this incommensurability makes it difficult to foresee how superintelligent AI would pursue optimization objectives. Indeed, these theorists argue that there are reasons to believe they will likely pursue such objectives in ways that will be harmful, even catastrophic for humans. Common arguments for such claims include the idea that superintelligent AI could pursue any type of final goal, not simply ones that we imagine and that to achieve these goals, superintelligent systems are likely to pursue a set of instrumental values like self-preservation or the acquisition of resources whose precise form may be difficult to predict, or even perceive (Bostrom 2014, pp. 107–114; Dung 2023, p. 7).

By assigning even very low subjective probabilities to catastrophic scenarios, in combination with estimates of all possible future human happiness, one quickly reaches extreme logical endpoints where focusing all intellectual resources on an unlikely future of total annihilation through AI becomes more important than investing in mitigating ongoing catastrophes such as climate change. “If we represent,” writes Bostrom, “all the happiness experienced during one entire life with a single teardrop of joy, then the happiness of these souls could fill and refill the Earth’s ocean every second, and keep doing so for a hundred billion millennia. It is really important that we make sure these truly are tears of joy” (2014, p. 103).

This strand of alignment thinking combines a probabilistic sensibility with a virtuosic imagination of scenarios, where the more unexpectedly an imagined system subverts an apparently benevolent goal, the more it is thought to reveal a general inability to control the behavior of superintelligent systems. Of course, allegories about the unintended consequences of our desires are one of the oldest forms of ethical fashioning; children still learn the moral of the story of King Midas.^10^ Similarly, the AI researcher Eliezer Yudkowsky imagines the development of an AI system that “optimizes for [human] smiles”—initially eliciting these desired behaviors through benevolent means. But he argues that a superintelligent system might discover disturbing ways to produce these behaviors more efficiently, for instance by injecting users with drugs. Even if the programmers are initially able to use their own ethical intuitions to forbid such *specific actions*, there would come a point where an superintelligent system could calculate that it could better meet its optimization objective by genetically engineering human brains to produce smiles, all while hiding these behaviors from its programmers (Yudkowsky 2016).

These scenarios produce a strange but rigorous form of asceticism, where, “each time we hear of a seemingly foolproof security design that has an unexpected flaw, we should pick up our ears. These occasions grace us with the opportunity to abandon a life of overconfidence and resolve to become better Bayesians” (Bostrom 2014, p. 130). They are also supplemented with empirical examples that imply that more severe unintended consequences lay beyond the horizon. One trope is to reference OpenAI’s work on a reinforcement learning system for the game CoastRunners, where the system learned a method of maximizing a reward score but in a way that contravened the designer’s intent or spirit of the game (Amodei et al. 2016; Clark and Amodei 2016; Dung 2023, p. 9). Likewise, cultural analogies used by the AI community, such as references to H.P. Lovecraft’s shoggoth monsters who precipitated the collapse of a fictional civilization, emphasize the alien nature of LLMs, one that may be hidden behind the domesticated “smiley face” of RLHF techniques, as Valentin Goujon and Donato Ricci show (2024, 11). In these diverse cases, the point again is to dramatize absolute divergences between benign intended goals or objectives set by programmers and extremely undesirable means or behaviors used by models to reach these ends.

We have stressed an ethical “minimalism” of alignment discourses; the peculiarity of an ethics that, because it has to deal with such a vast range of unforeseeable consequences, draws on values like “harmlessness” that seem capable of covering this expansive ethical territory but by the same token do not seem to provide a powerful source of ethical motivation. The existential sense of alignment might give more of a normative charge to this ethics. It certainly is not reticent about making life or death claims. Alignment researchers also frequently present the extreme technical-intellectual challenge of this form of ethics as a motivating factor. Yudkowsky, for instance, compares the level of difficulty to some combination of extremely prestigious engineering pursuits: building rockets, space probes, and cryptography (Yudkowsky 2016). More recently, the researchers Ilya Sutskever and Jan Leike have announced the need for “superalignment,” necessitating scientific breakthroughs that go beyond human forms of control in RLHF for systems in order to “supervise AI systems much smarter than us” (2023).^11^ Taking this logic to its conclusion, Bostrom advocates for “a strategy of deferred gratification”—in other words that philosophers think *only*, at least for a while, about the existential risks of superintelligence, before returning to traditional philosophical questions. This “more pressing challenge” is nothing less than the survival of the entire human species (Bostrom 2014, p. 256). Such an extreme one-sidedness, the subordination of all ethical and intellectual work to a single speculative control problem, sits at the furthest reaches of our absolutist pole of alignment.

In spite of this apparent extremity, we wonder whether these types of ethical claims end up subordinating a richer, positive set of values to the ultimately minimal imperative of human survival or existence. Certain critics of AI ethics speak of the subordination of “real harms” to “imagined” ones (Bender and Hanna 2023). This description fits, but by attending more closely to both technical and existential senses of alignment, it is possible to better account for these types of binaries: by fundamentally problematizing the ability to control model behaviors—whether this is due to the more precise technical nature of pre-training objectives in LLMs or the more extreme divergence between human control and “superintelligence”—an ethics of alignment stipulates and in some senses *enacts* a world in which the consequences of actions and the meaning of intentions or goals become more and more difficult to evaluate. What remains is the relatively weak possibility of retrospectively mitigating undesired behaviors or vague hopes for “new scientific and technical breakthroughs” (Leike and Sutskever 2023)—a *deus ex machina*. Where values are explicitly used is not so much to assert any kind of substantive control over a model, to guide us toward some meaningful *end*; indeed, problematizing intentions and objectives, alignment precisely precludes this type of ethical agency. In its place, it leaves feedback mechanisms to steer model outputs *away* from undesirable behaviors that are diagnosed after the models are used in social, which is to say normative, contexts.

## Defusing conflict, instrumentalizing ethics

Much of the emerging literature on alignment has a tautological character: “The goal of AI value alignment is to ensure that powerful AI is properly aligned with human values” (Gabriel 2020, 412). Who could disagree with such an intuitive idea? Our critical purpose in attending to the conceptual and technical particularities of alignment is to question such apparently self-evident positions, where the solution of alignment is deduced from the problem of powerful autonomous AI systems—that of control of norms and behaviors.

Our anatomy of alignment began with its basic logical senses that predate contemporary machine learning, forming its conceptual context. These include a relative sense of alignment in which objects are arranged in relation to each other and what we termed an absolute sense in which objects are oriented according to a fixed external reference point. Empirically, we later noted a proximate use of the phrase “value alignment” in a management discourse that emerged during the 1990s that sought ways of harnessing and harmonizing the individual interests of workers without relying on their forceful subordination to an organizational imperative (Senge 1990).

We then turned to the technical practices in machine learning that have been conceptualized in terms of the sense of alignment that predominates in AI ethics today. The cases we studied are characterized by a specific problematization of intentions or desires, where machine learning models, due to their statistical complexity or divergence from ordinary human cognition, behave in ways that subvert human instructions and intentions. Instead, models reflect ingrained norms and biases of society in ways that undermine the values of the context from which they emerge. Here, alignment refers to a spectrum of techniques that modify the behaviors of these systems, to guide their conduct by eliciting (rather than prescribing in any direct way) more “desirable behaviors”—a phrase that encapsulates its layered ethico-technical senses. In current practice, this usually means evaluating a sample of outputs in light of minimally defined ethical criteria and using these evaluations in an additional automated training process aimed at aligning—minimizing divergences between—model behaviors and the ethical expectations of the researchers and the companies.

We have shown that alignment mediates between two divergent forms of normativity that we see operating in machine learning: an “is” normativity of statistical structures identified in training data and an “ought” normativity involving human expectations and evaluations regarding model behaviors. The case of hallucination illustrates how these divergences between behaviors and expectations or intentions are identified and ethically problematized. The study of fine-tuning with RLHF shows how values are instrumentalized in automated processes designed to elicit norms that conform to the retrospective evaluation of undesirable model behaviors. The minimal ethical character of the values used in the fine-tuning process, such as helpfulness, harmlessness, and truthfulness, is striking in light of some of the maximalist claims about potential risks of AI systems made by prominent members of the philosophical community. We suspect that this minimalism stems from the fact that these values, like truthfulness, do not reflect prior, substantive ethical commitments by the AI community but are engineered post hoc to respond to the diagnosis of patterns of undesirable model behaviors, like hallucinations.

Finally, we connected these engineering practices with a more philosophical sense of alignment that grapples with speculative problems raised by the prospect of AI systems that would vastly outstrip human cognitive capabilities. We argued that this moves us from a relative, technically grounded sense of alignment between model behaviors and often implicit normative expectations towards an absolute, existential sense of alignment. In the existential discourse, the modernist belief in technological progress is replaced by the vision of a future catastrophe caused by technological progress. It is a form of discourse that doesn’t offer any shared social goal or substantive value toward which humans should strive with the help of technology. AI is construed as an irresistible force with an optimization logic of its own. All humans can do is to mitigate unanticipated risks through alignment techniques to contain the absolute power of AI that they have unleashed.

Although alignment has the intuitive allure of appealing to “human values”—itself a platitude whose universalism deserves scrutiny—as a way of exerting ethical agency over systems that otherwise could defy our control, in practice these values are less the substantive ends that a society chooses to live by than means used to train models to behave in ways that follow our intentions. Weber long ago noted that rational forms of ethical conduct tended to make life more predictable. Alignment works to achieve a similar form of predictability but in a technological situation where rationality has turned into its opposite: the unintended consequences and inexplicable behaviors of models. In so doing, it sidesteps the question of power and its asymmetries—who disposes of the massive amount of energy and data necessary to train models—through its reformulation in terms of shared moral values or preferences, a technical way of resolving or even pre-empting political conflicts. Machine learning mediates ambiguously between technical standards, values captured in the dataset and values introduced during fine-tuning, producing an apparent continuity between these levels that contributes to the appearance of consensus. This is not, however, the type of consensus produced out of a political struggle among a plurality of values, goals, and desires, to shape life and action in common.

Indeed, in its existential guise, alignment subordinates the political question of who has the power over models and how society should live with them in the here and now to speculative probabilistic arguments about the likelihood of human species extinction and vague, seemingly undebatable universal values; who could object to honesty, helpfulness, and harmlessness? Although the existential risk discourse gives ethical pathos and weight to the concept of alignment, it turns the problem of control of potential risks into an absolute horizon. Our purpose here is not to try and differentiate between real and imagined “harms” induced by AI—or, for that matter, between the equally unsatisfying poles of “doom” and “hype”—but rather to show more precisely how the logic of alignment leads to an instrumentalized ethics—to the use of technologies that incorporate values into their own optimization logic.

In elevating the control of risks to its core ethical concern, alignment disempowers rather than empowers society to make substantive valuations regarding ethical ends. In its aim to smooth divergences between undesirable behaviors and ethical expectations, to harmonize and ultimately identify “is” with “ought,” alignment, taken to its logical conclusion, forecloses the source of ethics and politics itself: the difference between life as it is and life as it could and should be. The ambiguous slippage between ethics and terms like “preferences” or “desired behaviors,” ubiquitous in the technical literature on alignment (Mu et al. 2024, 2; Ouyang et al. 2022, 6), speaks to this diminishment of the ethical to issues of management of behaviors and mitigation of risks. Whereas values can inspire ethical and political action, attempts to reach beyond the scope of what is currently possible, alignment reduces “desired behaviors” to correctives, to more predictable control of models. We hope our anatomy of alignment can jar us from complacency or a sense of inevitability. Understanding the contingent manner in which this ethical imperative connects very different technical, historical and normative levels may encourage others to invent more creative and critical tools to fashion values—not limited to correcting undesired behaviors but also to make life worth living in our technological milieus.

## Data Availability

We do not analyse or generate any datasets, because our work proceeds within a theoretical approach. One can obtain the relevant materials from the references below.

## Footnotes

1 The term “Deutungshoheit” refers in German to the authority claimed and the power exercised by a person or an institution within the political, economic, or social sphere to “interpret” something and thereby to evaluate it. This term emphasizes that norms become effective when they are supported by structures of power.

2 The observation that modernist rationalization can lead to irrational, even disastrous consequences has been made more or less persuasively by many social theorists (Beck 1992; Scott 2020). Our purpose is to describe a specific form of this tension that takes shape in the technical context of AI.

3 See, for example: Jaton (2021), Apprich et al. (2018), and Gitelman (2013). An important exception is the paper by Grosman and Reigeluth (2019) on algorithmic normativities, which discusses how algorithmic systems would have the ability to both follow and institute norms. While starting from a sociotechnical standpoint, which would account for the entanglement between technology and society, the authors end up considering algorithmic systems as autonomously norm-instituting. With the concepts of “is” and “ought” normativity, we instead operate beyond the human-machine distinction by considering dealing with norms as an always sociotechnically entangled endeavor. We focus on the ways in which machine learning systems either represent sociotechnical norms––by recursively contributing to the normalization or naturalization of these norms––or are the locus of the negotiation of more explicit norms and values, which we called “ought” normativity.

4 The unchallenged notion of the “naturalness” of data structures and data-label relations is deeply ingrained within the machine learning community and warrants a critical reading. As instances of the insistence on the naturalness of data structures, see Goodfellow, Bengio, and Courville (2016, p. 553), and more recently Arvanitidis, Hansen, and Hauberg 2021. This corresponds to what we have previously called an “artificial naturalism” (Campolo and Schwerzmann 2023).

5 This contextual aspect resonates in some ways with Helen Nissenbaum’s normative theory of privacy as “contextual integrity” (2004).

6 According to a recent review (Ji et al. 2023, p. 2, n.1), this more negative connotation is in fact recent, emerging roughly in 2020 as, we have stressed, generative language models were more widely adopted and used both in research communities and lay publics (Maynez et al. 2020). Interestingly, the term “hallucination” appears to have had a *positive* connotation in some earlier uses in computer vision, where it could refer to superresolution and other forms of image synthesis.

7 The alignment literature often expresses a fundamental distrust toward human judgment (Casper et al. 2023, p. 5), considered to be biased. In some recent approaches to fine-tuning, there is even a desire to get rid of the need for human evaluation altogether by imagining systems that would become less dependent on human feedback; such models would recursively evaluate and train on data they produce themselves (Gulcehre et al. 2023).

8 We thank Eva Weber-Guskar for making us aware of the similarities between alignment ethics and biomedical ethics.

9 We wish to thank Fintan Mallory for this reference. Bostrom, for instance, speaks of a principal-agent problem that arises with the creation of an artificial superintelligent agent (2014, p. 127).

10 This myth is nonetheless invoked explicitly by Stuart Russell (2019, p. 65).

11 As we were working on this article, Sutskever and Leike resigned from OpenAI, indicating deep tensions between parts of the research community focused on questions of existential risk or more generally “safety” and those primarily interested in economic returns from these technologies.
